# Congenital Insensitivity to Pain With Anhidrosis: A Case Report and Review of the Pertinent Literature

**DOI:** 10.7759/cureus.31019

**Published:** 2022-11-02

**Authors:** Muneer M Almutairi, Sadia Tabassum

**Affiliations:** 1 Pediatric Neurology, King Fahad Medical City, Riyadh, SAU

**Keywords:** case report, fever of unknown origin, anhidrosis, congenital insensitivity to pain, hereditary neuropathy

## Abstract

Congenital insensitivity to pain with anhidrosis, or hereditary sensory and autonomic neuropathy (HSAN) type IV, is an exceedingly rare neurogenetic disorder. Reported causes are homozygous or compound heterozygous loss-of-function mutations in the neurotrophic tyrosine receptor kinase 1 gene (*NTRK1*). Characteristically, patients with this disorder exhibit a complete diminution of pain and temperature sensations over the body disrupted sweat gland functioning, and variable degrees of sensation and cognitive impairments. We hereby present the clinical and neurophysiologic features of a 15-month-old boy with a homozygous frameshift mutation in c.1860_186insT. (p.Pro621Serfs*12) in the *NRTK1* gene, consistent with the diagnosis of congenital insensitivity to pain with anhidrosis.

## Introduction

Congenital insensitivity to pain with anhidrosis, or hereditary sensory and autonomic neuropathy type IV, is an ultra-rare neurogenetic disorder. The reported causes include homozygous or compound heterozygous loss-of-function mutations in the neurotrophic tyrosine receptor kinase 1 gene (*NTRK1*; #OMIM 256800) [1]. Typically, the clinical manifestations appear in early life, with a variable array of phenotypic features. Characteristically, patients with this disorder exhibit a complete diminution of pain and temperature sensations over the body with preservation of other sensory modalities, along with disrupted sweat gland functioning, and resultant impaired thermoregulation and recurrent hyperthermia. Nevertheless, the other autonomic parameters, including blood pressure, are functionally intact. As the disorder progresses, skeletal deformities (caused by recurrent traumas and fractures), cutaneous lichenification, corneal ulcerations, burns, and tongue ulcers (due to self-mutilation secondary to loss of pain sensation), as well as variable degrees of cognitive impairment are noted [2-6].

We report a 15-month-old toddler with congenital insensitivity to pain with anhidrosis. A retrospective chart review of the pertinent literature was conducted, and CARE (CAse REport) guidelines were followed.

## Case presentation

The child was born full-term via spontaneous vaginal delivery. There were no pre-, peri- or immediately postnatal events. Birth weight was 2 kilograms. He was the first child of healthy parents with first-degree consanguinity. There was no history of miscarriages or early neonatal deaths. Family history was not significant for hereditary conditions or neurologic disorders.

The child presented with fever and feeding difficulties at six days of life. The fever was remitting in nature and responded to antipyretics and cold packs. There were no associated respiratory symptoms, diarrhea, vomiting, skin rashes, joint swellings, and/or pain. Since the onset of these intermittent pyrexia episodes, he was evaluated thoroughly at several health facilities, but no diagnosis was confirmed. At eight months of life, the parents noted that he is failing to thrive and occasionally would bite his tongue, with an ulcer developing on the tip and right side of the tongue.

At this time, the global developmental delay also was noted. He was able to roll over and sit unsupported, but he could not pull to stand. He had a predominantly palmer grasp and was able to transfer objects from one hand to another. He understood simple commands with gestures and could only babble incomprehensible sounds. He knew his parents, smiled reciprocally, and experienced strangers’ anxiety. Due to the persistent tongue ulcerations, he required the extraction of his teeth on the same side.

On physical examination, his facial features were subtly distinctive with upslanting palpebral fissures, large protruding ears, a long philtrum, and a small mouth. He was microcephalic with a fronto-occipital diameter of 38.5 cm (2 standard deviations [SD] below the third percentile), and with failure to thrive. The weight was 5.7 kg (2 SD below the third percentile), height was 75 cm (at the third percentile), and he had a thin body build. The skin was generally dry with faintly colored sparse hair over the body.

The examination of the oral cavity revealed a partially healed 1.05 cm grayish ulcer on the lateral tongue, with an atrophic base and sharp borders. There were no exudates or discharges. Muscle bulk generally was reduced, and he had axial and appendicular hypotonia with normal power and brisk reflexes. Impaired pain and temperature sensations were noted. The remaining neurologic examinations, including the cranial nerves and cerebellar examination, were normal. Systemic examinations were unremarkable.

A thorough laboratory examination, including complete blood count, liver and renal function tests, and electrolytes, with a basic metabolic workup, was unremarkable.

Radiologic examination showed generalized osteopenia of all long bones. Brain magnetic resonance imaging revealed a mild reduction of the deep white matter and no structural abnormalities. A nerve conduction study showed evidence of moderately severe large fiber sensory motor axonal neuropathy with small fiber involvement, in view of absent sympathetic skin responses.

Blood samples of the proband were collected and sent for Whole Exome Sequencing after obtaining written informed consent from his legal guardian. The result of this came to be positive for a homozygous frameshift mutation in c.1860_186insT. (p.Pro621Serfs*12) in the *NRTK1* gene, consistent with the diagnosis of congenital insensitivity to pain with anhidrosis.

## Discussion

Hereditary neuropathy is a diverse group of disorders affecting the peripheral nervous system, with widely varying clinical presentations and genetic etiologies. They generally are divided into two main categories, including disorders in which: (1) neurons primarily are affected alone or as the prime element of the illness and (2) the neuropathy is merely an element of a more generalized or systemic illness [7,8].

Hereditary sensory and autonomic neuropathy (HSAN) represents a heterogeneous class of hereditary neuropathies in which sensory, and occasionally autonomic, neurons are the principal elements affected. These HSANs are subclassified as types I, II, III, IV, and V, with type I being the most and type IV being the second most common. Types IV and V are closely related and reportedly occur in the context of *NRTK1 *gene mutations. Type IV is characterized clinically by a triad of insensitivity to pain, anhidrosis, and intellectual disability, the latter of which is of clinical importance when differentiating types IV from V, since intellectual disability has not been reported in type V [9]. Furthermore, type V presents with milder degrees of anhidrosis and has been reported secondary to mutations in nerve growth factor beta (NGFB) gene. In addition, type IV is characterized notably by the preservation of lacrimal gland function as well as the fungiform tongue papillae, which differentiate this condition from type III HSAN [7,8].

Type IV, or Nishida syndrome, has been described as early as the 1900s by Dearborn as a case of “Pure Congenital Analgesia” and was reported for the first time 60 years later, after being thoroughly investigated, by Swanson. This disorder primarily develops as a consequence of the loss of a function mutation in the NRTK1 gene, or neurotrophic receptor tyrosine kinase 1, which is chromosomally located at 1q21-22 (*NTRK1*; #OMIM 256800). This gene is believed to have a crucial role in the development of nociceptive sensory and sympathetic autonomic neurons in the dorsal root sensory ganglia. This is evident histopathologically by the absence of small unmyelinated nerve fibers as well as denervation and atrophic changes of the eccrine sweat glands among patients with Type IV HSAN. It is clinically evident by the presence of anhidrosis, with unresponsiveness to pilocarpine in all patients with this condition [9-12].

Several types of *NRTK1* gene mutations have been reported, exceeding >50 mutations, including frameshift, and altered splicing sites mutations, missense, intragenic polymorphic sites, and deletions mutations, with frameshift mutations representing the most common type and (586 fs) being the most common site [13,14].

Our patient presented with significant anhidrosis, insensitivity to pain, and self-mutilating behavior (tongue ulceration) secondary to prolonged hyperpyrexia in the neonatal period and tongue ulcerations occurring after the eruption of the primary dentition in the infantile period. The syndrome was diagnosed by whole exome sequencing with supportive findings in the nerve conduction study.

Albeit being common, hyperpyrexia is a major cause of mortality among such patients. Reportedly, death occurs in 20% of cases by three years of age and 43% throughout life if combined with sepsis, bearing in mind the poor response of these patients to any stresses [4,15,16]. Skin and soft tissue infections are among the commonest reported infections in patients with CIPA, mainly due to Staphylococcal infections with a high rate of complications; thence, aggressive management and early use of local antiseptics are advised [17].

The therapeutic options in congenital insensitivity to pain with anhidrosis are limited, and management of such patients relies mainly on conservative symptomatic interventions such as keeping the patients well-hydrated and management of recurrent fevers due to its reported association with a high rate of mortality as well as avoiding injuries and trauma. Furthermore, the use of lubricant ophthalmic solutions and regular checks for injuries is of utmost importance in order to prevent any deleterious complications [17].

## Conclusions

In conclusion, congenital insensitivity to pain with anhidrosis is an uncommon entity of fever of unknown origin and should be considered in the context of self-mutilation in patients from areas with high consanguinity rates. To our knowledge, no targeted therapeutic agent exists for this disorder. Families should be counseled and trained to keep the child hydrated and avoid any injuries and stresses.
